# Supplementary material for: Current Clinical Landscape and Global Potential of Bacteriophage Therapy
Source: Viruses. 2023 Apr 21;15(4):1020. doi: 10.3390/v15041020 (PMC10146840; doi:10.3390/v15041020)
Supplement: Supplementary file 1 [file viruses-15-01020-s001.zip › viruses-2290802-supplementary.pdf]

Table S1. Research labs at universities and institutions that study phages.

Country	Institutions	
**USA**	University of California San Diego	Des Moines University
	Stanford University	Calvin University
	San Diego State University	University of Colorado
	UC Irvine	University of Alabama
	Johns Hopkins University	Old Dominion University
	University of Maryland	Virginia Commonwealth University
	U.S. Navy Medical Research Center	American Type Culture Collection (ATCC)
	Walter Reed Army Institute of Research	University of Florida
	J. Craig Venter Institute	Hanover College
	McDaniel College	Bethany College
	Georgia Institute of Technology	University of Central Oklahoma
	Emory University	Cornell University
	University of Northern Iowa	Queens College
	University of Pennsylvania	Rochester Institute of Technology
	University of Pittsburg	Texas A&M<br>Michigan State University
	Baylor College of Medicine	Duke University
**Canada**	University of Alberta	
	d'Herelle Reference Center for Bacterial Viruses	
	McMaster University	
	University of Waterloo	
	National Research Council Canada	
	Université Laval	
**Mexico**	Centra de Investigación y de Estudios Avanzados	
	CIBA-IPN	
	Universidad Autónoma de Ciudad Juárez	
**Brazil**	University Center SENAI CIMATEC	
	University of São Paulo	
	Universidade Federal do Pará	
	Universidade do Estado do Amazonas	
**Venezuela**	Universidad Simón Bolívar	
**Ecuador**	Universidad de las Fuerzas Armadas – ESPE	
**Chile**	Pontificia Universidad Catolica de Valparaíso	
**Kenya**	Jomo Kenyatta University of Agriculture and Technology	
	University of Nairobi	

Table S1. Research labs at universities and institutions that study phages.

**Egypt**	Center for Microbiology and Phage Therapy	
	Zewail City of Science and Technology	
**Nigeria**	Plateau State University Bokkos	
**Ethiopia**	Jimma University	
**Australia**	Australian Phage Biobanking Network	Flinders University of South Australia
	University of Sydney	Macquarie University
	Massey University	Monash University
	University of Adelaide	La Trobe University
	University of Western Australia	
**Singapore**	National University of Singapore	
	Nanyang Technological University	
**South Korea**	Bacteriophage Bank of Korea	
	Seoul National University	
**Israel**	Hebrew University and Hadassah Medical Center	
	The Volcani Institute	
**India**	Indian Institute of Science	National Centre for Veterinary Type Cultures
	Panjab University	SRM Institute of Science and Technology
	Bhabha Atomic Research Centre	Karnatak University Dharwad
	ICMR-NICED	University of Madras
	Vellore Institute of Technology	Amrita Vishwa Vidyapeetham
	Cochin University of Science and Technology	
**China**	Wuhan Institute of Virology	
	Fudan University	
**Pakistan**	University of Punjab	
	Hazara University	
	University of Haripur	
**Nepal**	Tribhuvan University	
**Indonesia**	University of Jember	
	National Research and Innovation Agency	
**Thailand**	Silpakorn University	
**Iran**	University of Isfahan	
**Japan**	Rakuno Gakuen University	
**Turkey**	Mikroliz Therapeutic Bacteriophage Bank (MTBB)	

Table S1. Research labs at universities and institutions that study phages.

**Philippines**	University of Santo Tomas	
**Germany**	German Collection of Microorganisms and Cell Cultures	
	Research – UFZ, Forschungszentrum Jülich	
	Planck Institute for Terrestrial Microbiology	
	Helmholtz Center for Environmental	
**Italy**	Experimental Zooprophylactic Institute of Lazio and Tuscany	
**Netherlands**	TU Delft	
**Belgium**	Queen Astrid Military Hospital	
	Ghent University	
**United Kingdom**	University College of London	University of Warwick
	University of Manchester	University of Sheffield
	Canterbury Christ Church University	National Collection of Type Cultures (NCTC) Blower Lab
	University of Exeter	Durham University
	University of Leicester	Quadram Institute of Bioscience
**Georgia**	Eliava Institute of Bacteriophages	
**Spain**	A Coruña Hospital, Centro Nacional de Biotecnologia (CNB-CSIC)	
**Switzerland**	Zurich University of Applied Sciences	
	University of Lausanne	
	Zürcher Hochschule für Angewandte Wissensch (ZHAW)	
**Sweden**	Lund University	
**Ireland**	APC Microbiome	
	Teagasc	
	Cork Institute of Technology	
**Poland**	Hirszfeld Institute of Immunology and Experimental Therapy	
	University of Wrocław	
**Portugal**	University of Aveiro	
**Russia**	Federal Service for the Oversight of Consumer Protection and Welfare	

* Laboratories and bacteriophage banks listed are based on the online phage directory (available at: https://phage.directory) and do not include biotechnology companies or start-ups. This table may not be all inclusive.
